# Cryopreservation and current legal problems: seeking and selling immortality

**DOI:** 10.1093/jlb/lsad028

**Published:** 2023-11-09

**Authors:** Alexandra Mullock, Elizabeth Chloe Romanis

**Affiliations:** Law, The University of Manchester, Manchester M13 9PL, UK; Durham Law School, Durham University, Durham DH1 3LE, UK; Harvard University, Petrie-Flom Center for Health Law Policy, Biotechnology, and Bioethics, Cambridge, MA 02138, USA

## Abstract

Cryonics, the ‘freezing’ of the human body after death in the hope of reanimation in the future, remains a remote possibility, and yet it is becoming a more popular choice. There has been much academic discussion of the ethics of cryopreservation; however, the legal problems have received little attention. There are, however, several potential current conflicts that might arise, as was illustrated by the case of *JS* in England, in which a 14-year-old girl who sought cryopreservation against her father’s wishes. In the USA, there have been disputes within families about cryonic preservation, and between cryonics organizations and loved ones of the deceased when there is negligent preservation. Cryopreservation raises questions concerning the law on death and posthumous interests, property in the body, contract law, and (potentially) negligence. We argue that, in the absence of proper regulation, cryonics organizations may be able to exploit the dying and dead. The potential legal problems that we have identified in relation to the law in England and Wales demonstrate that the law is ill-equipped to protect the interests of the dead and their next of kin.

## I. INTRODUCTION

Waking the dead after cryopreservation remains science fiction. While freezing human cells and tissue is well established in medicine—particularly freezing gametes or embryos for fertility treatment—life after death via cryopreservation remains impossible. Despite the state of the science, people are opting for this technology and entrusting their ‘remains’ to cryonics companies, and interest in doing so is increasing.^1^ Individuals can arrange for either their entire body or their head to be frozen after death, and thereafter preserved with the hope that reanimation (and cure) is eventually possible. The question of whether such a hope will ever be fulfilled is not our concern and this article does not attempt to engage with unknown futures and science fiction. Rather we are concerned about the current legal uncertainty over preserving the dead in this way and the potential for legal conflict this raises for the living relatives of the deceased in the present. In this paper, we consider the legal uncertainties in the jurisdiction of England and Wales.

An example of conflict over cryopreservation was seen in *Re JS,*^2^ in which a 14-year-old-girl with a rare form of terminal cancer successfully sought a court order to allow her body, after death, to be cryonically preserved without the approval of her estranged father. While this case set no precedent approving or encouraging cryonics,^3^ questions about the way the process would be handled prompted Jackson J to conclude that proper regulation of cryonics was needed.^4^ As *JS* illustrated, the current law is struggling to accommodate the cryonic process, the interests of those preserved and their relatives. The conflict in *JS* reveals how those with an interest in what happens to the body of a deceased relative may seek to prevent cryopreservation. Moreover, because the process and maintenance of cryonics involves an ongoing obligation to preserve the body of the deceased, whose legal rights died with them, questions arise, and are addressed in this paper, over the nature of any legal obligations to the next of kin and importantly, who has the legal right to possess the preserved body or head in the event of a dispute.

While the ethics of cryonics have been considered in some depth,^5^ the legal questions have received less attention. Perhaps this is unsurprising. Conway takes the view that so few people in the UK are choosing cryonic preservation that regulating to address such rare needs is ‘not such an urgent task’.^6^ However, we believe that examining the issues to better inform the debate before regulation becomes a more urgent task is a worthwhile exercise. Some people are already using this technology, and the potential for legal problems and conflict is significant. Many of the legal issues we explore in this article are speculative, but such an investigation is necessary to consider what may be problematic about cryonics, and to consider potential legal solutions.^7^ Furthermore, because of the importance attached to respecting the wishes and the remains of the dead, and the logistics of preserving and storing the dead in this context, cryopreservation raises particularly sensitive and potentially disturbing issues. Such issues require thorough consideration before a court is compelled to resolve a dispute concerning cryopreservation.

There is nothing to prevent individuals in England and Wales from choosing cryonic preservation after death, provided they can pay for it and make the necessary arrangements to be frozen and stored at a cryonics organization. There is, however, somewhat of a legal vacuum when it comes to the potential legal problems or conflicts between interested parties surrounding cryonic preservation. Consequently, it is important to examine problems that have arisen—in *Re JS* and in the USA—and to speculatively consider foreseeable legal problems related to use of technology in advance of such conflicts materialising in the jurisdiction of England and Wales.

In this article, we first consider how the initial arrangements are conducted with the relevant organizations and the challenges of ensuring that cryopreservation ensues, particularly in England and Wales. These complications may arise while the person intending to become a cryon is still alive/dying and thus is a legal person. We then discuss ‘conflicts about cryopreservation’—namely, what happens where family members oppose cryopreservation (even though favored by the dying/dead individual), or where the state has requirements for bodily treatment/investigation after death, or, if a problem arises with the process immediately after death. We then consider ‘conflicts in preservation’—namely, disputes between the dead/their next of kin and cryonics organizations where there is a failure to cryonically preserve a body, or the process is in some way negligent. All these disputes are complicated because they often occur once the person who had wanted to be preserved is no longer alive, and thus no longer a legal person. Finally, we consider ‘disputes after preservation’—these are primarily questions of who, in the event of a dispute, has the right to possess, or perhaps ‘own’ a cryopreserved body when that body (definitively no longer a legal person) effectively becomes a *chattel*. While the general rule is that the body is not property, we argue that cryonic preservation could transform human remains into property. While the family initially has a strong claim in deciding what happens to the body in terms of how the body is disposed of, once the cryonic organization takes possession and exercises skill in preserving the body, the issue of *ownership* becomes unclear.

As *Re JS* illustrated, people may opt for cryopreservation as a way of dealing with or processing their death, perhaps as a means of obtaining some comfort and hope about the end of their life. Our analysis demonstrates that this choice makes them incredibly vulnerable in several ways. We highlight how, without proper regulation in a number of areas, cryonics organizations are able to exploit the dying and dead, and potentially also create significant legal problems, with associated emotional distress, for the family of the deceased person.

## II. CHOOSING CRYOPRESERVATION

Since its inception in the 1960s, cryonics has been regarded by many in the scientific and medical community as ‘a grievous form of quackery’.^8^ Recent advances in the understanding of vitrification,^9^ however, together with anticipated developments in nanotechnology,^10^ have been held out by various organizations providing preservation services as proof of principle for cryonics. The cryopreservation process needs to happen quickly. The process must not begin until after a person is legally dead,^11^ but it must be done *as soon as possible* after death. Because of autolysis (tissue decomposition), which begins immediately after death, Alcor advises that ‘[c]ryonics procedures should ideally begin within the first one or two minutes after the heart stops, and preferably within 15 minutes’.^12^ The object is to intervene before decomposition causes significant damage. Alcor advise that they will have a team ‘on standby’ for around 7 days before they believe a patient may die.^13^ The process of cryopreservation involves dehydrating the body to remove water from all the body’s cells, flushing the body with a cryoprotectant substance (a kind of human ‘antifreeze’ as an organ preservation solution) before the body is cooled on ice and placed into liquid nitrogen for storage.^14^ This process of preservation, while still experimental, is a vast improvement from initial attempts in the 1960s and 1970s because it has been shown to prevent tissue damage. Far less is known about how the body could ever be reanimated from cryostasis. Organizations offering cryopreservation claim that reanimation is foreseeable based on what we already know about nanotechnology.^15^ Presumably, if preservation is a sound technique, we can take it as read that there would be no urgent requirement to perfect defrosting techniques: the condition of the frozen (ex-) person will not change.

The USA leads the way in cryonics with both the Alcor Life Extension Foundation and the Cryonics Institute at the forefront of both ‘caretaking’ and research into process and technique. Both organizations were founded in the mid-1970s and each claims to have over 150 ‘cryons’^16^ in their care.^17^ Trans Time in San Francisco is also known for providing suspension and caretaking to both domestic and international clients.^18^ There were previously a greater number of organizations providing or assisting clients with cryonic preservation arrangements in the USA, but a number has ceased accepting new members.^19^ Despite being primarily practiced in the USA, cryopreservation is in demand across the globe. Consequently, cryonics organizations have emerged that assist individuals in other countries accessing suspension arrangements in the USA either by subcontracting with Alcor/Cryonics Institute/Trans Time to preserve clients or offering advice about aspects of arrangements such as the financial aspects. There are also organizations providing Cryonics services outside of the USA, such as KrioRus in Russia.^20^

These organizations offer several services, none of which is cheap. In *JS*, the cost of cryonic preservation was highlighted as approximately 10 times that of an average funeral,^21^ although this estimation may be on the low side. Alcor charges around $200,000 dollars for whole body preservation, but also offers neurocryopreservation (in which only the head is preserved and stored) for $80,000.^22^ If only the head is preserved, it is presumed that the headless body will ordinarily be buried or cremated in accordance with the preferences of the deceased and/or the next of kin. For those in the UK wishing to be preserved, an organization called Cryonics UK is able to provide assistance.^23^ They describe themselves as a nonprofit organization, and in 2018 they successfully appealed a decision to remove their charitable status.^24^ This organization can arrange to attend the death of the individual wishing to be preserved. They have an ‘ambulance’ in which to attend to begin cryopreservation and arrange transport to the cryonics organization responsible. In *Re JS*, it was reported that the hospital agreed beforehand that Cryonics UK would be given advance notice of death, as the process is not possible without sufficient warning. Jackson J commented on this process:

Evidently where the subject dies in hospital, the cooperation of the hospital is necessary if the body of the deceased is to be prepared by the volunteers (*who are not medically trained*).^25^ This situation gives rise to serious legal and ethical issues for the hospital trust, which has to act within the law and has duties to its other patients and to its staff. ^26^

Following the death of JS, distressing evidence from the hospital emerged:

The voluntary organisation is said to have been under equipped and disorganised, resulting in pressure being placed on the hospital to allow procedures which had not been agreed. Although the preparation of JS’s body for cryonic preservation was completed, the way in which the process was handled caused real concern to medical and mortuary staff.^27^

Reading the information provided by Cryonics UK on their website provides a clue about why the process is both distressing to those who witness it, and legally and ethically contentious. Among other interventions, the dead patient is ventilated so that their heart pumps blood, and this is done by non-medically trained amateur volunteers. Drugs are administered to the body, and the cooling process begins. This involves treating a dead body in an unusual way, with a process that seems to fall in a novel space, somewhere between a medical intervention and a process usually done by an undertaker. Measures to preserve a corpse are not usually done at the hospital, and obvious concerns emerge when an amateur team of cryonic enthusiasts enters the hospital to perform this work. The hospital has no legal obligation to support this process, just as they would have no obligation to allow undertakers to perform their work in the hospital mortuary. It appears to be a matter of good will on the part of the hospital, not an arrangement that a potential cryon could necessarily rely on, and so people who expect to die in an NHS hospital would need to persuade the trust to accommodate their wishes.

One of the tensions arguably evident when Cryonics UK attended the death of *JS* is the way they treated the body of the deceased. In *JS*’s case, this caused concern among staff, but this might also have caused distress to the family and friends of the deceased if present. Even where the family is supportive of the decision to be preserved, the reality of witnessing the arrival of the cryonics team to process and take possession of the body might be distressing at a time when the bereaved are particularly vulnerable. They might wish to spend some time with the deceased before the body is taken, but the cryonics process demands that preservation begins immediately. While there are similarities with the way that an undertaker will take possession of the body to prepare it for burial or cremation, the evidence from *JS* suggests that the urgency required, together with the nature of the cryonic interventions, may be potentially distressing for the next of kin. Some of the bereaved might also feel upset because the traditions of death (a funeral with burial or cremation) are not followed, although it would of course be possible to have a funeral or memorial event with no body, or indeed an event to mark the burial or cremation of the headless body (if only the head has been preserved). This potential for distress, however, may be managed if the family feels positive about the process, experiencing hope of eventual reanimation for the deceased, rather than the usual finality of death.

From the perspective of cryonics organizations, the preserving process is not seen as readying the body for disposal because their ethos involves rejecting the clinical and legal fact that preserved individuals are dead.^28^ Instead they purport to *treat* people rather than dispose of them, and they refer to them, as *patients*. One of the leading cryonics organizations, Alcor, claims cryonics is not an interment method or mortuary practice, but rather ‘an extension of critical care medicine’.^29^ The Cryonics Institute observes that it ‘do[es]n’t put in this effort for a “dead person”’, but for a patient perceived as stabilized indefinitely, and who can eventually be saved.^30^ For our purposes, because ‘saving’ the deceased is currently impossible, it is, for the time being and the foreseeable future, legally irrelevant. In *Re A,* an infant was found to be ‘dead for all legal, as well as medical purposes’ after a diagnosis of cessation of brain-stem function.^31^ In *Bland,* Lord Goff stipulated that

‘[r]ecent developments in medical science have fundamentally affected…previous certainties. In medicine, the cessation of breathing or of heartbeat is no longer death….This has led the medical profession to redefine death in terms of brain stem death i.e., the death of that part of the brain without which the body cannot function at all without assistance’.^32^

These two cases elucidate that legal and medical definitions of death align; specifically, they confirm that legal definitions of death are dictated by medical definitions. The Academy of Medical Royal Colleges stipulates that

‘[d]eath entails the irreversible loss of those essential characteristics which are necessary to the existence of a living human person, and thus the definition of death should be regarded as the irreversible loss of the capacity for consciousness combined with irreversible loss of the capacity to breathe’.^33^

Given this medical definition, ‘as a matter of law, it is the case that brain stem death is established as the legal criteria in the United Kingdom’.^34^ Brain stem death is treated as a threshold medical diagnostic test: ‘[a]ascertaining death requires the application of clear clinical guidelines’.^35^ A series of neurological tests, undertaken by two senior doctors,^36^ for brain stem death are thus used to make a legal, as well as a clinical, determination of death. We regard a ‘cryon’ as legally dead since they have no brain stem function. The clinical tests—designed to determine if there is an irreversible loss of consciousness and inability to breathe—would surely conclude that the patient was dead (there would be no response to stimuli, no unaided breathing, etc.) Furthermore, such function is not recoverable, even considering the theoretical possibility of cryonics—the clinical determination would have to be that consciousness and breathing are not recoverable. While we accept that there may be room for debate about whether a small theoretical possibility of recoverability in future might mean a diagnosis of death is precluded, there is (at the time of writing) no evidence to indicate that a person who is regarded as medically dead could ever be recovered. Consequently, the law that regulates disposing of the dead requires examination to assess how the option of cryopreservation fits alongside traditional methods of disposal or whether it needs to be wholly distinguished to be regulated in a different way.

## III. CONFLICT ABOUT PRESERVATION

Moen positions the option of cryonics alongside traditional methods of disposing of the dead —burial or cremation—as an equivalent option with the bonus that there is a ‘non-negligible’ chance of reanimation.^37^ Put that way, cryonics might be seen as a benign choice. However, we suggest that the issues raised by cryonics are too complex and contentious to view it in simple terms of another option for disposing of the dead. In this section, we examine the conflicts that might arise about whether a body should be cryopreserved. The legal issue in *Re JS* arose because JS was a child and one of her parents objected. For adults with capacity wishing to be cryopreserved, no one would have legal authority to prevent them from arranging cryopreservation, but their wishes could not be guaranteed to be followed. We will consider the legal issues arising if an individual wants to be cryopreserved, but their next of kin (spouse or registered civil partner) and/or their family are not supportive. We also consider instances in which an individual wishes to be cryopreserved but may be prevented from doing so for legal reasons.

Unlike some countries whose law insists that property is shared in a particular way,^38^ English law provides the utmost respect for the wishes of the dead to dispose of their property however they wish, but there is no legal means to ensure that one’s body is disposed of in a particular way.^39^ English law is clear that funeral instructions are not legally binding.^40^ As Cantor explains;

[w]hile a decedent may have a theoretical right to dictate the fate of his or her property or to control or heavily influence the disposition of personal remains, the interests of the corpse tend to be less robust when they conflict with the needs and wants of living persons. The descendants are alive and pushing their interests as opposed to the silent an immobile decedent.^41^

Consequently, although there is human rights authority that indicates that the views of a deceased person regarding their funeral arrangements and the disposal of their body ‘must be taken into account’,^42^ there is no mechanism for compelling those responsible for arranging the funeral and body disposal to do as the deceased wished.

Looking back for authority on the disposal of the dead, we see that certain rights have been established, but these are restricted only to a basic right to have one’s body disposed of. From the early Victorian period, a common law right to a ‘Christian burial’^43^ was established. Subsequently, a broader principle that it is in the public interest to dispose of the dead on the grounds of human decency and public health was recognised.^44^ Responsibility for disposal falls upon the Executor/s appointed by any will of the deceased and if the deceased died intestate, the duty falls to the family, with a hierarchical approach.^45^ Any spouse sits at the top of the hierarchy, with children second, followed by parent/s, then siblings. In the absence of a spouse and any living close relatives, the search for a relative to dispose of the deceased expands to seek any distant family able to fulfill this role and if none can be found, the duty of disposal falls upon the local authority.^46^ The clear aim of these legal obligations is to avoid human corpses rotting in public view, with only a broad right to have one’s body disposed of subject to the preferences of the person/s obliged to fulfill that obligation.^47^

This law might have changed to enable people to be sure that their funeral wishes were followed if the Law Commission’s proposal, in their 13th program of law reform (2017–2020), had been followed. The Commission indicated that it would ‘seek to provide greater certainty that a person’s wishes about what happens to their body after death are respected’.^48^ If this change is eventually adopted, greater certainty may be achieved. However, if the law is reformed as recommended, it is not inevitable that any new law would necessarily include protection for all options for disposing of bodies, so it could be that greater postmortem respect for the wishes of the deceased would be restricted to options that the law approved of, such as in France,^49^ where burial or cremation are mandatory.

Unlike France, the UK has no legislation insisting that the dead must be disposed of in a particular manner. Accordingly, as was confirmed in *Re JS,* there is nothing to prevent individuals from choosing cryopreservation after death, provided they can pay for it and make the necessary arrangements to be frozen and stored at a cryonics organization.^50^ But what happens when there is family opposition to the choice made by the deceased? To make the necessary arrangements for cryopreservation, it is helpful if the individual has persuaded all executors and their next of kin that cryopreservation is desirable.

The conflict that led to the case of *JS* perfectly illustrates the potential for trouble even before the individual planning their own preservation has died. JS’s youth exacerbated the problem and led to a parental dispute about what should happen upon her death. JS needed the court to set aside her estranged father’s (initial) opposition before cryopreservation could go ahead, and subsequently to establish that her mother had the sole right to determine what would happen to her body. In contrast, an adult will not require permission to seek cryopreservation and will be free to make the necessary arrangements. As we have noted, however, there is no law to ensure that the wishes of the putative cryon are respected.

Dying (and dead) people are not in a position to organise their own timely postmortem preservation with the certainty they might wish for. This highlights the need to have supportive next of kin, or perhaps an appointed representative,^51^ to instigate the cryonic arrangements immediately, with the technicians on standby waiting for death. In *Re JS*, the court determined that the father could not interfere with his daughter’s wishes, and the authority lay solely with the mother, who was willing to ensure that her daughter’s wishes were respected by facilitating the necessary arrangements. People planning to undergo cryonic preservation after death are often concerned that their next of kin may veto any plans. This fear is apparent in online discussion forums about what has been termed the ‘hostile wives phenomenon’. Such discussion has claimed that there are instances of wives/girlfriends preventing their husbands/partners from inquiring about cryonics at all, or contracting for cryonics services, and/or forcing the lapse of membership to cryonics organizations.^52^ While this is simply one report that suggests how conflict can occur, a US case demonstrates a clear example of this variety of problem.

The case of deceased American baseball player, Ted Williams, illustrates how conflict within families can arise.^53^ After Ted’s death, his son arranged for cryopreservation at Alcor in the USA with the agreement of his sister, Claudia. But Ted also had an older daughter, Barbara, who disagreed with her younger half-siblings, and sought to have her father cremated. Confusion over what Ted wanted arose after several friends reported that he had said he wanted to be cremated, leading Barbara to petition the court to have Ted’s body thawed and returned from Alcor. Eventually, after other (financial) disputes between the siblings were resolved, Barbara agreed to drop the case, leaving Ted’s body at Alcor. While we do not know precisely why Barbara agreed to drop the case, the fact that there was a simultaneous financial dispute between the siblings alerts us to the potential for financial interests to conflict with plans for cryopreservation. Indeed, the context of the ‘hostile wives phenomenon’ reveals how the inheritance hopes of putative beneficiaries, who wish to avoid the high cost of cryopreservation, can derail the wishes of the deceased. This was also a factor in *Re JS* because the father was concerned that he would be obliged to fund his daughter’s preservation. Such financial concerns could, however, work in favor of a wealthy person seeking cryopreservation if they decided to use inheritance incentives, perhaps via a trust, to persuade their next of kin to arrange cryopreservation.

A dead body is not property and therefore cannot be bequeathed in a will.^54^ As Conway explains, ‘the right to possession is purposive and transient; it is a custodial right that exists solely for disposal’.^55^ Hale J (as she then was) observed that ‘there is very little modern authority on the use of this power and none at all on its use’ where there are conflicts between interested persons.^56^ In such cases, the Court cannot direct how a deceased person is disposed of (among the options), but can declare who has the power to do this among any people contending an interest in doing so.^57^ Thus, in a case of family conflict over the disposal of body of the deceased, the solution lies in determining who has the right/responsibility for disposing of the body. As outlined above, any executor/s of the deceased are obliged to dispose of a body and so an individual planning their cryopreservation will presumably choose their executors on the basis that they can be trusted to arrange cryopreservation. Problems might arise if that trust was misplaced. As Cantor suggests, the only solution might lie in careful planning with a clever legal mechanism to help ensure their wishes are followed:

‘Some testators aware of the hazard of survivors’ resistance to prescribed wishes, have tried to set up a self-enforcing mechanism for having their disposal wishes honoured. One testator provided in his will that if his next of kin failed to cremate his body his assets should be diverted…to the local cremation society’.^58^

If the deceased died intestate, it falls to the next of kin (spouse first, children second, and so on) to arrange the cryopreservation. The potential for conflict is obvious. If those in conflict had a seemingly equivalent right to determine the means of disposal—for example, two or more siblings—it would fall to the court to resolve the dispute. If the conflict meant that the cadaver was not frozen in a timely fashion, the wishes of the deceased (to be preserved for future reanimation) would be compromised because of tissue decay even if the court did subsequently rule that the relative in favor of arranging cryopreservation on behalf of the deceased should be permitted to do so.

Several cases within the jurisdiction have involved similar family disputes. For example, in *Hunter v Hunter,* a son succeeded in having his father cremated rather than buried, against his late father’s stated wishes, on the basis that the deceased had experienced a surprising and very late religious conversion (to Catholicism) at a time when his mental health was questionable.^59^ The court was evidently persuaded that as a devout protestant for most of his life, the most significant and durable wish of the deceased had been to be cremated, not buried in a Catholic grave. Consequently, they allowed the son to arrange cremation in accordance with what the court deemed to be the most compelling evidence concerning the wishes of the deceased. Religious conflict is common ground for disputes about disposing of the dead. In *Saleh v Reichert*,^60^ the husband and father of the deceased disagreed over whether the deceased should be buried in accordance with Muslim faith or cremated. The husband succeeded in having his wife cremated because the evidence showed that was her wish. Our final example of this type, *Burrows v HM Coroner for Preston*,^61^ was not a matter of religion but rather a dispute between a mother and an uncle about what the deceased wanted. The uncle had been the main carer for the deceased, a 15-year-old who died by suicide while in custody. The uncle succeeded in persuading the court that his nephew wished to be cremated rather than buried, as the mother had wanted.

Like the decision in *Re JS*, we suggest that these cases show that, although the wishes of relatives are relevant, the court is primarily concerned with respecting the (stable and most enduring) wishes of the dead. Therefore, we would anticipate that if a dispute over cryopreservation arose, provided that the conduct of the deceased clearly indicated that they wished for cryopreservation, the court is likely to support that desire. If the wishes of the deceased were followed immediately after their death but there was a subsequent attempt to regain custody of the corpse to bury or cremate the deceased (illustrated by the disagreement between the children of Ted Williams), the law becomes more interesting and contested. We will discuss this in the last section (conflict after preservation).

The final point in this section relates to legal requirements outside the family. If we assume that the person wanting to opt for cryopreservation does have a supportive family willing to ensure that preservation takes place, the legal formalities of death might prevent preservation. Every death occurring in England and Wales must be registered within 5 days by a ‘qualified informant’ (usually a family member who assumes responsibility) under the Births and Deaths Registration Act 1953. If the cause of death is known and a doctor has issued a Medical Certificate of Cause of Death (MCCD), the registrar will issue a death certificate and disposal of the body can go ahead. With traditional means of disposal (burial or cremation) this means that cremation or burial must not take place until the death certificate is obtained. Under certain circumstances, however, an MCCD cannot be immediately issued:

- If no doctor saw the deceased during the course of the final illness and the cause of death is not known.
- If the death was sudden, unexplained, unnatural, because of violence, neglect, or other suspicious circumstances.
- If death occurred during or shortly after surgery/general anesthetic.
- If death was caused by industrial accident, disease, or poisoning.
- If death occurred in prison, police custody, or other state detention.^62^

In these instances, no death certificate—and thus no disposal—will be immediately possible because the death must be reported to the Coroner, who may request that a postmortem autopsy is carried out to establish the cause of death. Indeed, even if there is no autopsy there might be problems with accessing the body to perform cryopreservation, e.g. where the person has died in state custody. The need for an autopsy is a rare eventuality, but because it could thwart any attempt at cryopreservation, it requires our consideration. The Coroner has the power to *order* an autopsy if death is violent, unnatural or suspicious, for example, if a drug overdose or suicide is suspected. In these circumstances, the postmortem examination is required by law,^63^ and so it is not possible for the next of kin to avoid the autopsy to arrange cryopreservation.^64^ The law allows only an appeal against a decision *not to* conduct an autopsy,^65^ for example, if the family believe the death was suspicious but the Coroner believes otherwise, and so there is no legal avenue to prevent an autopsy if a Coroner has ordered one. The Coroner also has a temporary right to possess the body until the inquiry is completed.

Most people die in circumstances not requiring a Coronial inquiry and we might presume that individuals seeking cryopreservation would take all possible steps to avoid this; however, we are all potentially vulnerable to an unexplained death, so this aspect of the law presents a further potential obstacle. If we envisage a scenario where the cryonics team attend a death expecting to begin working on the body immediately, but the doctor/s in attendance are insisting that the death is unexplained or suspicious and so should be reported to the Coroner, the conflict would be palpable. However, the law is clear that the doctor/s, and subsequently the Coroner, have the power to prevent the family (and thus the cryonics company) taking possession and disposing of the body in this scenario.

We are not aware of any cases concerning a conflict between a family wishing to facilitate cryopreservation and the formal necessities of registering and investigating death in England and Wales, but there have been conflicts associated with religious values.^66^ For example, interfering with a corpse might be seen as desecration, or immediate burial might be demanded by certain religions. Conway discusses how such cases could perhaps give rise to a human rights violation under article 8 (private and family life) or article 9 (freedom of religion) of the European Convention.^67^ In relation to article 8, the same might be said about taking possession of a body for the purposes of cryopreservation but this would be a weak claim set against the obvious importance of investigating unexplained death. Moreover, from a practical perspective, while any such conflicts were playing out, the window of opportunity for immediate cryopreservation would be closing.

## IV. CONFLICT IN PRESERVATION

In this section, we examine the conflicts that might arise once the person intending to be cryonically preserved is legally dead and all relevant parties agree that the body should be cryopreserved. Before we go into the analysis, there is the broader question of whether a cryonics question is enforceable at all.^68^ This is a question with a normative element: are people and should people be free to contract about cryonics? Generally speaking, the law is especially supportive of freedom to contract;^69^ there are very few instances in which English law will intervene with this general principle. Some examples of limits placed on freedom of contract as a matter of public policy are where recognition of the contract would amount to commodification,^70^ or would put one group at a systematic disadvantage.^71^ Some of the problems we describe below in imagining that a cryonics contract is enforceable might illustrate how there may be public policy reasons not to recognize a freedom to contract about cryonics. For example, because the body could become property of a cryonics company or there would be limited suitable remedies in the case of a cryonics company failing to hold up their end of the bargain. We will not discuss the normative matter of whether cryonics contracts are enforceable further in this paper, though this is something that should be explored elsewhere. The contribution to the literature we make here in exploring the consequences of enforceable contracts in this context may assist in work addressing the normative question.

Assuming there are not thought to be public policy reasons to exclude a freedom to contract in this context, a cryonics contract would be prima facie enforceable. English law has been willing to enforce provisions of a contract that are specific about what happens if one party dies—presumably the law would also hold that contracts about what happens after one’s death would be enforceable. Parties can make contractual arrangements about what happens in the event of death, and in principle there is nothing stopping a promisee from making it a term of the contract that in the event of their death their executor can ensure that the promise is fulfilled. Seemingly then, the ‘dead’ have contractual rights to goods and services—in the sense that these rights that can be ‘inherited’ and enforced by their estate. There is an interesting question about the nature of that estate in how long ‘the estate’ exists for (it usually just ceases to exist after all the property has been dispersed)—pertinent in the cryonics problem as will become apparent later in this article.

In English law, the common law maxim *action personalis moritur cum persona* (an action dies with the person concerned) does not apply to contract. Consequently, some breaches of contract are possible to recognise, and remedies can thus be enforced, postmortem.^72^ Contracts for personal services, however, are sometimes known to be frustrated by death. Frustration—which enables parties to an agreement to be discharged from their obligations—occurs when performance of the contract is rendered impossible by an unforeseen event that neither party is responsible for. Death will frustrate a contract in cases where a promisor dies and their estate cannot perform their end of the bargain.^73^ If a person who is unique to the contract dies and can no longer perform their promise, it makes sense that the contract ends.^74^ However, in a case where a contract contains a provision as to what happens in a given set of circumstances, there will normally be no frustration in those circumstances because the contract will determine the rights and duties of the parties involved. Where a contract is for cryonics services, there is nothing to stop the services (preservation) from being performed because the person who contracted for the services is dead. The doctrine of frustration is inapplicable since the contract directly deals with death; the contract is not frustrated since death does not prevent performance (in fact, it is necessary for performance). The contract is thus prima facie enforceable on the individual’s death by their estate/executor (though the mechanism of enforceability is debatable, this is discussed below). In what follows, we will *assume* that the person intending to be cryonically preserved has a legally binding contractual arrangement with a company providing preservation services to provide close analysis of some of the problems that could result in instances of conflict.

### IV.A. Failure to Preserve

What happens if a cryonics organization fails to perform the promised preservation services? Or, if a family refuses to hand over a corpse for preservation? These are problems that might arise about the enforceability of contractual arrangements regarding cryonics as well as what remedies might be available. While these situations might seem far-fetched, both have already materialised and resulted in complex legal disputes in jurisdictions outside of England and Wales.

#### 1. Contractual Breach

In 2015, Laurence Pilegram, aged 90, died of a suspected heart attack near his home in Southern California, USA. Pilegram had contracted with Alcor long before his death to have his entire bodily remains cryogenically preserved ‘no matter how damaged’.^75^ He had arranged for his life insurance to cover the $200,000 sum—paid directly to Alcor on his death—for entire body preservation. Directly following his death, Alcor was made aware, and arrangements put into motion for cryopreservation. However, the plan went wrong;

‘Alcor dispatched two of its technicians to the morgue, where they removed Pilegram’s head, packed it on ice, and drove it back to Scottsdale. The rest of his remains were cremated and mailed to his son Kurt in Montana. When Kurt demanded to know why his father’s whole body hadn’t been preserved, he received conflicting accounts from Alcor, according to court records. First, the company said Laurence’s body had decayed beyond saving. Then, it claimed he hadn’t kept up with his yearly $525 membership dues. Finally, it suggested the technicians didn’t want to wait for the permit necessary to transport a full body across state lines.’^76^

This case illustrates some of the many circumstances in which a cryonics organization may attempt to claim that they were unable to preserve a body, leading to a family challenging a failure to preserve. Equally, one can imagine a different scenario—one where a cryonics organization seeks compensation from the family for the breach of contract where they have failed to provide a body to preserve or the payment for so doing.

Assuming there was a valid arrangement for preservation in this case, the cryonics organization that failed to preserve may have breached the contract. While a more unlikely scenario in circumstances where a family failed to ‘hand over’ a person’s body for cryopreservation where that individual had (before their death) contracted for a company to preserve them, this may equally be in breach of the contract on this logic. It might be suggested that a cryonics company is unlikely to pursue action in such a case because of concerns about negative media attention;^77^ however, they have incentives to act in that they are both committed to the practice and aiding those who are also believers and because of the lost potential profit where a consumer contract goes unfulfilled. Furthermore, challenging families in this way might be used as a way of deterring other families from seeking to prevent preservation. The company would be correct in pointing out that the agreement from their perspective is also enforceable (where the arrangement is valid)—since the services they are contracted to provide (and to be reimbursed for) are specifically supposed to happen after death.

In circumstances like the Pilegram case, Alcor could attempt to argue that other potentially unforeseeable events prevent the contracting cryonics organization from performing their contractual obligations and being thus frustrated. This might happen in several (admittedly unlikely) circumstances; for example, if the state does not release the individual’s body for preservation because a postmortem is required, or the individual dies in the wilderness, far away from any medical support, or their body is badly damaged in a fire. Such situations, if not provided for expressly in the contract, may have the effect of frustrating the agreement and discharging them of their obligations. The same, of course, is true in some circumstances where a family may choose not to allow the cryonics organization access to the body because, for example, an unforeseeable event occurs leaving the body in a state that they believe the person would not want it to be preserved; for example, if the body is badly damaged by a fatal accident. In the Pilegram case, it is possible to construe either the decaying of the body beyond saving because of the specific circumstances of the death, or the complications in obtaining a permit to transport the body across state lines as frustrating events—assuming they were not foreseeable. Thus, there are potentially foreseeable situations in which a frustrating event might occur.

For the purposes of the next section, however, we will consider the contract to be enforceable (not frustrated) and consider whether this is specifically enforceable or enforceable with the help of damages. A cryonics contract is clearly a consumer arrangement, as a cryonics organization is ‘acting for purposes relating to that person’s trade, business, craft or profession’ to perform a service for an individual.^78^ The Consumer Rights Act 2015 (CRA 2015) is clear that ‘every contract to supply a service is to be treated as including a term that the trader must perform the service with reasonable care and skill’.^79^ There has clearly been a breach of this condition in a situation like that of Pilegram, and so we now consider the available remedies.

#### 2. Remedies for Cyro Contract Breach

The question of what remedy a dead person’s estate would be entitled to in the event of a breach of a valid contractual agreement to preserve is a fascinating one. Where a cryonics contract does not explicitly specify the remedy for contractual breach, the most likely outcome of failing to cryopreserve a body is likely to be either a price reduction as per the CRA 2015,^80^ or rescission (putting the parties in the position they would have been in if the contract had never happened).^81^It is possible to return any monies paid for preservation, and to cancel any subscription for future storage costs, but it is impossible to demand performance, or to compensate for any ‘loss’ (as we will illustrate later in this section). With only these two remedies available, this is highly advantageous for cryonics companies whose contracts are effectively risk-free, while consumers are, in contrast, unable to effectively guarantee that the services they have contracted for will be performed, or that a sufficient remedy is available to them in the event it cannot be.^82^ A cryonics contract that stipulates a remedy for nonperformance could rectify this perceived imbalance—but the bargaining power between cryonics organization and consumer is such that this is hard to imagine being of significant benefit to the consumer. This, we argue, is a strong justification for the need for specific regulation of cryonics arrangements. Some specific designation of remedy to protect the consumer seems necessary.

Where a service does not conform to the contract, consumers have a range of options including (but not limited to) CRA 2015 specific-remedies (repeat performance,^83^ and price reduction),^84^ and general contractual remedies like compensatory damages,^85^ specific performance, ^86^ or rescission.^87^ A consumer cannot require repeat performance of the service if it is impossible,^88^ which it would be if there was a failure to properly cryopreserve a body in the first instance. Once a body is destroyed by other means, such as cremation, there can be no preservation. It also seems that there can be no *successful* preservation if there is any real passage of time since the death. If a person has contracted for cryonic preservation with some future chance of reanimation, we might assume that they would expect to be preserved in a timely fashion. If the organization failed to fulfill that expectation, the consumer may be entitled to a price reduction.^89^ Where there is no preservation at all, a complete refund of any price paid may be sought, unless the cryonics organization is able to argue that they sent a team to preserve and it was not possible for reasons outside their control.^90^ Furthermore, in circumstances of partial cryopreservation, like that of Pilegram, the return of the difference between complete and partial preservation seems like a reasonable price reduction for the services actually rendered. However, there may also be a strong argument that the full sum should be returned because the service that should have been performed was not*.* Complete and partial(neuro) cryopreservation are very different. In cases where a contract is breached by a failure to cryopreserve at all, it is likely that both of these remedies would prove insufficient—repeat performance is not possible, and price reduction may not adequately compensate the innocent party for what may be perceived as a loss in the event of incomplete/partial performance of the contract on the part of the cryonics organization. This brings us to the applicability of general contractual remedies.

The principal remedy for contractual breach is compensatory damages (payment awarded to the victim of the breach to compensate for loss sustained because of the breach). Damages should, as far as money can, place the injured party in the same position that they would have been in had the bargain been performed.^91^ The first step of ascertaining what compensation is awarded in circumstances of breach is to identify the loss the innocent party has sustained as a result of the breach. English law recognizes actionable losses as purely financial losses (estimated as expectation damages),^92^ but in some instances it will also recognize nonfinancial loss where there is actionable distress. What is the loss where a body is not cryopreserved as per a contractual arrangement?

This is a difficult question since nonfinancial loss is likely to be of more importance to the consumer. The value of a cryonics contract to a person before they are dead is much greater than its ‘market value’ (the literal cost of the preservation and storage). The Court has recognized that people often contract specifically for, and on the basis of, nonfinancial values, such as relaxation when booking a holiday.^93^ The value that an individual places on such factors, where this exceeds the objective market value of the goods/services in question, has some recognition.^94^ The Court has recognized the necessity of damages where the fruit of the contract is pleasure or peace of mind to one party and this does not materialise.^95^ Equally, damages can be awarded for physical inconvenience and discomfort caused by the breach and mental suffering directly related to that inconvenience’,^96^ and ‘sensory’ inconvenience (affecting sight, touch, hearing, smell, etc.).^97^

While the idea of their body not being preserved would—presumably—be very distressing to an individual who contracted for cryonics before their death, there is obviously no sensory experience of mental distress resulting from non-preservation after death. Indeed, the value the person placed, before dying, on being cryonically preserved will almost certainly have been significant, but there is no frustration to this value where that individual is not aware/able to experience it. The notion of harm after death is seemingly legally and philosophically incoherent, since the dead have no enforceable rights in common law,^98^ and ‘there is no one who can be harmed at the point that any wrongful setback of interests occurs’.^99^ Damages are recoverable at the date of the breach^100^—at which point the dead person suffers no recognizable legal harm in their own right.

In some circumstances, English contract law has recognized the ‘loss of a chance’ as a form of actionable loss for which compensatory damages can be awarded. Most of these cases relate to circumstances where the claimant lost a chance at securing (more) funds^101^ and where, on the balance of probabilities, it is likely that they would have acted in a way to realise the chance were circumstances different.^102^ Though the person who is the subject of failed cryopreservation is dead, might the law recognize that they have ‘lost the chance’ to be reanimated—and thus alive—again? A creative court might address these questions as a means of identifying the appropriate remedy, for example, by analogizing the breach of the cryonics contract to the successful loss of a chance precedents. In *Chaplin v Hicks*, an actress was awarded substantial damages after having been selected as a finalist in a beauty contest but informed too late to participate, thus missing out on a 12/50 chance of winning a prize.^103^ The defendant had argued that the chance of winning the prize had turned on so many contingencies that it would be impossible to conclude that there was any assessable value to that loss of a chance. The Court of Appeal, however, determined that the law is very capable of recognizing ‘the existence of a liability which is incapable of being estimated’.^104^ Williams LJ explained ‘the fact that damages cannot be assessed with certainty does not relieve the wrong-doer of the necessity of paying damages for his breach of contract’. ^105^ Similarly, Moulton LJ concurred that ‘the very object and scope of the contract were to give the plaintiff the chance of being selected as a prize-winner, and the refusal of that chance is the breach being complained of and in respect of which damages are claimed as compensation’.^106^ On this logic, it might be argued that since the *chance* of being reanimated, no matter how remote, was at the heart of the contract, any action of a cryonics company that completely eradicates that chance should be properly conceptualized as a breach of the nature of the denial of that chance. However, the Court was clear that ‘there are cases, no doubt, where the loss is so dependent on the mere unrestricted volition of another that it is impossible to say that there is any assessable loss resulting from the breach’.^107^ Furthermore, ‘[i]t is obvious, of course, that the chance or probability may in a given case be so slender that a jury could not properly give more than nominal damages’.^108^ In our cryonics case, we take it that the loss is so dependent on scientific speculation that is yet to be shown to be possible that, at best, nominal damages could be awarded. While it seems conceptually difficult to accept that some chance has been lost in these cases, the reality is that the contract speaks to what both contracting parties think of as ‘a chance of future life’. A breach of performance means to the parties that there is no possibility whatsoever of the chance described in the contract being fulfilled. This might, therefore, be argued to be a breach of contract. But, if it were, the possibility of reanimation is incredibly speculative, and for people already preserved, likely to be completely impossible, and thus any damages awarded would be nominal.

In the event that a claimant (a deceased person’s estate) could prove actionable loss resulting from contractual breach, there would be a problem in *quantifying* that loss so that an amount of damages can be awarded to compensate. We have noted that if this claim of successful breach resulted from loss of a chance, damages would likely only be nominal. English law does not generally recognize the possibility of punitive damages for breach of contract.^109^ So, in quantifying damages there is no account taken of the contract breaker’s behavior in failing to perform their end of the bargain,^110^ but it is a matter of compensating for the loss incurred directly. Where losses are nonfinancial, and therefore not calculated as an expectation loss (to put the party in the financial position they would have been in had the breach not occurred),^111^ this is a more complex matter. Such damages in contract will be nominal,^112^ and are at the Court’s discretion. Thus, even though the cost of the breach may feel invaluable to third parties to the contract, any damages would be minimal.

Kurt Pilegram has described his lawsuit against Alcor as having the objective, among other things, of obtaining punitive damages for the ‘chronic anxiety and stress’ he experienced because of discovering that this father had not been preserved as per his agreement with Alcor.^113^ However, it is notable that it is the deceased person and their estate that was party to the contract that is breached. Thus Kurt’s loss may appear not to be actionable in English contract law by virtue of the traditional application doctrine of privity.^114^ In some circumstances, judges have carved out exceptions to privity in domestic circumstances to mean that a party to the contract, in this case the deceased’s estate, can recover for damages in respect of a third party’s loss, in this case family members being distressed by contractual breach.^115^ The exception, since codified in the Contracts (Rights of Third Parties) Act 1999, is deemed important to prevent a lacuna in the law in which one party to the contract cannot be held accountable for their breach of contract because the loss that results is to a third party.^116^ In *Jackson v Horizon Holidays*, Lord Denning expressed that such an exception was ‘the only way in which a just result can be achieved’.^117^ This might be considered pertinent in the cryonics context, where the contracting party will be dead by the time services are (not) rendered. In *Jackson,* the third parties were compensated because the holiday that had been contracted for, on their behalf, was a disaster. In the judgment, Lord Denning gives other examples of cases where services are contracted for on other people’s behalf, e.g. a coach rental or a restaurant booking for a group—where it would seem unjust not to recognize the harm to the third parties that might result from breaches like a coach not turning up, or a restaurant failing to serve food.^118^ While it may be possible to analogize a benefit to a close next of kin, such as a parent or a spouse, in another’s preservation—for example, they might enjoy some peace of mind that they have fulfilled their loved one’s wishes, or even benefit from feeling like there may be a chance they could live again. However, these are all examples where the third parties are integral to the object of the contract; a family member books a holiday for the whole family, a person books a restaurant for a group to eat, etc. A cryonics contract might be distinguishable because the service in the contract is directly for the person contracting for preservation. Third parties can enforce contractual provisions where the contract expressly enables them to do so;^119^ they need to be easily identifiable from the contract, for example, ‘identified by name, a member of a class or answering a particular description’^120^ and so, ‘next of kin or family member’ would be sufficient so third parties may also be able to enforce contracts this way. However, a cryonics organization (that has more bargaining power in the arrangement) is able to exclude this liability.^121^ It is more common in these circumstances for harmed third parties to pursue a more direct action in tort where their specific harm may be recognized.^122^

### IV.B. ‘Negligent’ Preservation

The discussion of contractual conflicts pertained to situations where there is a failure to preserve *at all*, or there is a partial preservation. What happens in those circumstances where there is negligence, for example, an attempt to preserve that falls below the standard expected? This could involve a failure to preserve the whole body, or where the preservation is not performed correctly, such that the body suffers significant damage rendering reanimation in future *even more* unlikely or impossible. Or worse, what if the negligent custodianship of the body led to it being used or treated or displayed in a way that was both disrespectful to the deceased and distressing for the family? It is possible that there could be an action brought in negligence by parties not in a contractual arrangement with the cryonics organization—family members—on the basis that there was a failure to take reasonable care in preserving and storing the body, which caused psychiatric harm to the claimant. Here, we examine the elements of the tort of negligence in the cryonics context where the action is brought by someone other than the deceased as a secondary victim.

This area of law has traditionally been unreceptive to claims of a novel nature to avoid opening the gates to the expansion of negligence in English law. Secondary victims of psychiatric injury can only succeed if they have witnessed the negligent misdeed inflicted upon a close relative or the immediate aftermath,^123^ and consequently suffered a clinically recognisable psychiatric illness beyond grief; ‘the law cannot compensate for all emotional suffering even if it is acute and truly debilitating’.^124^ For example, a parent who witnesses a child being seriously injured or killed by a negligent driver and consequently suffers psychiatric injury, such as post-traumatic stress disorder (PTSD) or clinical depression, would almost certainly succeed. Such a tragedy would satisfy the ‘time and space’ proximity requirement; that the claimant was present at the scene of the accident.^125^ This rule demands that secondary victims must witness the accident or the immediate aftermath with their unaided senses.^126^ For secondary victims not present at the scene, not even the most serious psychiatric harm caused by heinous negligence would enable a successful claim. Thus, it seems highly unlikely that a negligent failure to preserve the deceased would lead to a successful negligence claim because relatives would presumably not be present at the scene or the aftermath of the negligent preservation or other negligent treatment of the deceased.

However, unlikely it is that a relative could be present at the scene or the immediate aftermath of negligent preservation, the facts of *Re JS*—where the cryonics team began their work in the hospital—show that it is not impossible. Consider, for example, if a cryonics team mistakenly decapitated the deceased because they wrongly thought only head preservation had been arranged, as occurred in the Pilegram case. If the family was in the vicinity of the preservation process, they might witness the body immediately afterwards and become distressed by the mistake. Viewing the headless corpse of the deceased, and/or the head, the bereaved may suffer trauma that later develops into PTSD. It would be reasonably foreseeable that such negligence could harm a person of ordinary fortitude (who here would be the relative).^127^ The negligent act in question would presumably qualify as an unreasonable breach of the expected standard for cadaver preservation, and so provided the harm (PTSD) was clinically verified, the causative link could feasibly be established. Although in *Alcock*,^128^ the identification of the dead in the mortuary several hours after the Hillsborough tragedy was not deemed sufficiently proximate, in the scenario outlined here the trauma is not related to death but rather the wrongful decapitation so it would be possible to distinguish from *Alcock*. In *Galli-Atkinson v Seghal*,^129^ a mother who witnessed the badly injured and distorted body of her deceased daughter in the mortuary following a road accident succeeded, which supports the case for some flexibility on this issue. Such a scenario could therefore arguably lead to a successful claim. If successful, the claimant would receive damages for their pain and suffering and any associated losses, for example, loss of earnings if PTSD led to the claimant being too ill to work.

The final point in this section relates to the global nature of cryopreservation. This paper does not explore conflict of laws across different jurisdictions as this would add an additional layer to already complex problems, but this is an issue that could be further explored. We also focused on England and Wales given this is the jurisdiction in which we are both trained. It is important to acknowledge, however, that as the leader in cryonics, the US legal picture is also relevant and so it may be important that claims for negligent mishandling of a corpse causing ‘emotional distress’ have succeeded. As this is a matter for State Law rather than Federal law, approaches vary, but one successful claim was seen in Maine,^130^ when the severed leg of a deceased man was unexpectedly returned to his son along with other personal effects. Similarly successful claims have been seen elsewhere, such as New York and West Virginia, whereas other states, such as Illinois, have sought to limit the scope for such claims based on proximity rules not dissimilar to those restricting liability in English law.^131^ Thus, it appears possible that some US state courts might be sympathetic in the event that a Cryonics organisation negligently mishandled a corpse, leading to emotional distress for the relative/s.

## V. CONFLICT AFTER PRESERVATION

In this final section, we consider cases of potential conflict that might occur after the process of cryopreservation has been completed. This might include disputes about who ‘owns’ a cryopreserved body. We demonstrate that there is the possibility that a cryon might be regarded as a unit of property, and thus disputes about what happens to a cryopreserved body may come down to claims of possessory interest.

### V.A. ‘Ownership’ Disputes

What happens if two individuals lay claim to a cryon? These situations are not hard to imagine—consider again the case of the Williams family dispute in which two siblings wanted to keep their father preserved, and a third wanted the body given to them for cremation. It is also plausible to imagine circumstances in which, once a body is preserved, a cryonics organization would be reluctant to return it to family members since they may be benefitting financially from its continued storage (if they have a subscription model).

There is very limited opportunity to claim ownership of the cadaver of a deceased relative as the general rule is that there is no property in the body.^132^ The only exception can arise if the body, or tissue from the body, has been the subject of work and skill which means that the tissue can be differentiated from its natural state. In *Kelly*, the court approved the ‘work or skill’ principle determined in an earlier case, *Dobson*.^133^ This derives from an Australian case in which the ownership of a two-headed fetal corpse was disputed:^134^

[W]hen a person has by *lawful exercise of work or skill* so... that it has acquired some attributes differentiating it from a mere corpse awaiting burial, he acquires a right to retail possession of it, at least as against *any person not entitled to have it delivered to him for the purposes of burial*.^135^ (Our emphasis)

Two questions are therefore relevant here; first, would the cryopreservation of the corpse (or the head of the corpse), count as ‘work or skill’ to confer property rights to the cryonics organization who carried out that work? Second, *if* there is such a property right because cryopreservation is viewed as constituting ‘work or skill’, which differentiates the remains of the deceased from mere remains, would that trump any right enjoyed by a person entitled to dispose of those remains?

*Kelly* does specifically indicate that some preservation techniques are sufficient to transform tissue into property,^136^ and thus we suggest that if this principle was applied the cryonics organization could successfully gain a property right and become the owner of the preserved corpse. Grubb notes that ‘there is no doubt that the anatomical specimens in Kelly had undergone considerable change by the expenditure of skill and were preserved’.^137^ This was in stark contrast to the factual determination made in the earlier case in *Dobson*,^138^ in which the Court found that the ‘work/skill’ exception did not apply to a brain that had been removed from a corpse (and not returned to the family). The court found that ‘the minimal work or skill’ that was applied to the biological material (the preservation of the brain in fluid) was 'insufficient for the application of skill exception’.^139^

In *Kelly,* Rose LJ stated that body parts that ‘have a use or significance beyond their mere existence’ might be property for the purposes of theft.^140^ This context is important; the legal analysis was only focused on finding a sufficient right to support a charge of theft and not the type of dispute imagined here. A cryonics organisation would see a cryon as having an important purpose—storage in the hope of future reanimation—and it is easy to see how it could be naturally thought of in terms of possession. For example, we suggest that it would or should be a crime (a theft, rather than a kidnapping) to steal a cryon from a storage facility. That said, while the legal analysis is related to the criminal law, the decision embraces more broadly the principle that there can be property in the body and does not exclude this principle from being applied in other areas of the law. In *A v Leeds Teaching Hospitals Trust,*^141^ Gage J firmly concluded that ‘in my judgment the principle that part of a body may acquire the character of property which can be the subject of rights of possession and ownership is now part of our law’.^142^ He explains that in *Kelly* Rose LJ had explicitly endorsed general principles from the Australian case (*Doodeward),* which had been deliberately broad. In *Doodeward,* Griffith CJ had noted that ‘it is not necessary to give an exhaustive enumeration of the circumstances under which such a right may be acquired’, continuing (as we quoted above) to explain that where a person lawfully exercises work or skill on a human body giving it attributes differentiating it from a mere corpse awaiting burial then they acquire rights of possession.^143^ Cryopreservation is an extreme form of preservation involving scientifically sophisticated processes, which significantly change the cadaver from its natural state.^144^ It is not a simple matter of deep-freezing the corpse, which, upon thawing, would convert to a natural (pre-preservation) state. We suggest that, following the reasoning from these cases, cryopreservation would factually be seen as making considerable changes in preservation (see our earlier description), such that there is work and skilled performed, transforming a cryon from a dead body (or head) into a unit that can be regarded as property, and not merely a body for disposal, for the purpose of possession. There is, however, the possibility that we should view the reasoning from *Kelly* and *Dobson* as entirely inapplicable in this context because cryons are distinguishable from the kinds of tangible products made from human remains that were envisaged in the Court’s reasoning. The kind of work and skill the Court had envisaged involved the transformation of (part of) the human body so that it becomes a fundamentally different object—and something that cannot be reverted to its previous state because of how it has been transformed. By way of contrast, cryonics might be seen as a transformation that does indeed result in a significant change in the condition of the body but there is, at least within the contemplation of the parties, the hypothetical possibility that this transformation is later ‘undone’ (and, in fact, it is intended that it is undone).^145^ If Kelly and Dobson are not applicable, how might of ‘ownership’ be resolved?

The case of *Yearworth v North Bristol NHS Trust*,^146^ which involved a failure to successfully preserve the sperm of several men undergoing treatment that might damage their future chances of fatherhood, might have some relevance here. Indeed, this case is illustrative of the fact that there need not be a strict dichotomy between property and not property when it comes to ‘ownership’ of bodily materials. The anticipated ‘sticking point’ in *Yearworth* was the fact that sperm is not property^147^. However, adopting a malleable interpretation of the ‘no property’ rule, the Court held that in *such circumstances* the sperm should be regarded as property. Arguably, this case supersedes *Kelly* and *Dobson,* and thus the need to establish that a cryon is property, rather, it could be argued that it is necessary to establish that it is something that, *in the particular context* is capable of being *owned* for the purposes of recognizing loss if there is a lack of reasonable care.^148^ The Court of Appeal specified that, to determine if something was capable of being owned, required a contextual examination: there ‘must be into the existence or otherwise of a nexus between the incident of ownership most strongly demonstrated on the facts of the case’.^149^ In *Yearworth*, the fact that the sperm had been ejaculated with the sole object ‘that it might be used later for their benefit’ and thus needed to be stored in the interim.^150^ A cryon might be contextually analyzed in the same way—there is a contract for the generation of a cryon (from a dead body) for potential future use and it needs storage for that purpose. The owners of the sperm in *Yearworth* were the men who produced it because they had ‘absolute negative control over it’,^151^ as a result of relevant regulation determining that they could specify how the sperm *cannot* be used.^152^ There is a question in the cryonics context, however; does anyone have absolute negative control over the cryon? This might depend on the way the contract for preservation and storage is constituted (e.g. does the executor of the estate reserve the right to terminate storage?). For the purposes of our continued analysis, we consider that the cryon is (at least contextually) a tangible object capable of being ‘owned’.

The second question we raised above, about whether property rights trump rights of disposal, however, casts some doubt on our conclusions here because any property right is conditional upon there being no one entitled to take possession of the body ‘for the purposes of burial’.^153^ The answer here would depend upon the facts of the claim and the status of the person seeking to take possession for the purposes of disposal. But assuming that the cryonics organization had taken initial possession of the cadaver in legitimate circumstances, with the authority of a legally entitled person (executive of the will or next of kin), we propose that the ‘work or skill’ principle may usurp any claim by another person attempting to take (back) possession of the deceased for disposal. At first glance, it would seem likely that a property right would usurp any entitlement of disposal which confers only a *temporary* right of possession. Also, the cryonics organization could argue that they had disposed of the body (though if they were to successfully make this argument this would preclude any claim of a proprietary right in a cryon) in a manner that satisfied the joint human decency and public health objectives within this area of law. As noted by Peter Gibson LJ in *Dobson,*^154^ if families have a ‘right of possession’ it would be solely for the purposes of burial or cremation. Such a right is recognized in the common law but is enforceable only in equity.^155^ This might mean that the entitlement to dispose of the body of the deceased, which the claimant was seeking to rely on, had evaporated because disposal had already occurred at the point at which storage in a cryonics facility had begun. In 2012, a widow petitioned an English Ecclesiastical Court to exhume her husband’s buried cremated remains to have them buried elsewhere, in a place with more flexible rules about what might be placed graveside.^156^ The case is of some utility, because—although in this case the remains are not property—the decision of the Court to prevent the removal of the remains for disposal elsewhere illustrates the temporal nature of the right to disposal. This case illustrates how the conclusion, that disposal occurred at the point that the preservation process on the body was completed and the cryon was placed into storage, could plausibly be reached. Thus, any argument—based on a right to disposal—would be limited if the person who was entitled to dispose had consented to cryonics. It may be argued, however, that the conclusion was based on the importance of the permanence of burial,^157^ and cryonics may factually be distinguished as a state not intended to have permanence.

If, however, the claimant was an executive or relative with a genuine entitlement to possess the corpse for disposal purposes, and that entitlement was afforded no respect in the process, for example, if they did not hand over the corpse for preservation, their duty to dispose affording a right to possession might be construed to trump the potential property rights conferred by the work done. This scenario might arise in a case with two individuals—once again the sibling example is apt—enjoying commensurate rights of disposal. If one sibling acted without the approval of the other in transferring the body over to the cryonics organization, then although the cryonics organization had acted legitimately because they had (or believed that they had) the authority to take the body, there would be potential for a subsequent conflict between their property right and the disposal right of the claimant seeking possession of the body. In such a scenario, we suggest that both parties might have an arguable claim. As Grubb explains, the effect of *Kelly*^158^ is that rights to possession can be asserted over a dead body where work and skill has been expended on the body and the other circumstance ‘of much longer provenance, concerns the right of executors and administrators to the body’.^159^ So what happens when these two circumstances are in direct conflict in a cryonics dispute? It would fall to the court to determine whose right was more powerful.

Furthermore, if the cryonic-preserved body was likened to property there might be a claim made by the family, not on the right to possess a body in order to dispose of it, but rather that they own the preserved body because they are the executors of the estate that had contracted for the preservation. For example, if they wished the body to be moved and maintained at an alternative cryonics organization because they preferred it. The Courts in both *Doodeward* and *Kelly* were clear that it was the person who performed the work and skill who has ‘possessory claims’ in preserved remains, however in neither of these cases is there a contract, which makes things materially different. One comparison is where a person takes their deceased pet hamster to be stuffed by a taxidermist; the taxidermist performs the work and skill, but ultimately it makes sense to say that the person who *commissioned* the taxidermy owns the product/deceased hamster. This example, however, is distinguishable. The hamster did not become property once stuffed, it already was a chattel belonging to the owner (and the taxidermist possesses it under bailment). In the case of a cryon, however, there was no chattel that could be possessed until *after* the preservation (*if* the ‘work and skill’ principle is accepted). Still, there could arguably be significance in the commissioning that leads to the creation of something that can be possessed that might be determinative of ownership. Once again *Yearworth,* (as discussed above) might have some relevance because preserved sperm was considered proprietary material;^160^ the sperm belonged to the person who emitted it, despite the fact that it is the work of a laboratory technician in preserving it that makes it into something that can be possessed and that can be potentially useful, and therefore, of value in the future. Similar logic might be deployed to suggest that it is the executor of the estate that owns a cryon.

If this is the case, can the person/s seeking possession (likely the remaining next of kin) decide that they want to dispose of their property? As a rule, individuals are, of course, entitled to destroy their own property. An individual (in the UK) can lawfully opt to destroy their frozen gametes, or even frozen embryos, so why not their deceased frozen relative? The obstacle would be the competing ownership claim of the cryonics company founded on the ‘work and skill’ principle.^161^ The idea of owning their ‘customers’, however, seems to jar with the ethos of cryonics organizations, who appear to see themselves as the ‘custodians’ rather than the ‘owners’ of the preserved.

One argument in favor of regarding a cryon as a chattel owned by a Cyronics company is that cryonics organizations might be more likely to uphold a person’s wishes in favor of cryopreservation. However, there should also be concerns about exploitation if companies that have been paid to preserve dead people are also deemed to ‘own’ the deceased. If a cryonics company was able to establish that they ‘owned’ cryons, they would presumably be able to destroy them without recourse on the part of others. It would be rather unusual for a contract to enable one party (A) to require another person (B) to preserve or store property that belonged to B.^162^ With significant legal uncertainty over the conflicting claims that could emerge from this type of dispute, the court would face a challenging exercise interpreting and applying these doctrines to a novel problem. While we have argued that the ‘work and skill’ principles examined above might potentially convert cryonically preserved human remains into property, we also note that any dispute about ownership, possession or control of a ‘cryon’ would raise unique issues. The right to own a sample of human tissue is significantly distinct from any right to own an entire preserved body, or even merely a head, in this context. As Jackson J intimated in *Re JS*, public policy considerations over the purpose and legitimacy of cryonic preservation requires careful consideration.

Ultimately, since such a case would enable the court to choose between competing rights, we suggest that evidence about what the deceased would have wanted would likely become influential in the court’s reasoning and the question of ‘who owns a cryon?’ might become a complex matter of examining who, for policy reasons, the law believes will better uphold the wishes of the deceased. We agree with McGuiness and Brazier that the wishes of the dead do matter and should be respected because to do otherwise is detrimental to the living. Although we cannot say that the dead are harmed when their premortem wishes are ignored, there is a strong argument that they deserve to have their wishes (over what should happen to their body after death) respected.^163^

### V.B. Reanimation Disputes

Our final point considers a problem that is less current that the other issues we have explored, but it is an issue that requires some thought because of the likelihood for problems in the future. Given that there is no guarantee of reanimating a preserved body (and it may never be possible), the private contractual arrangement that a person has with a cryonics organization seems to be for preservation and storage, and not for reanimation. Many cryonics contracts guarantee storage for 100 years and explicitly do not guarantee reanimation.^164^ This has the potential to cause conflict in the future, in the event that reanimation becomes scientifically possible. We will not explore this in detail, since we do not believe this to be a current legal problem, but we note it for interest. What happens if a cryonics organisation, for whatever reason, does not want to attempt to reanimate a corpse, but the executor of the estate of a deceased person who has been cryonically preserved seeks to ensure that a reanimation attempt is made? There might be cause to recognize an implied contractual term within (what is effectively) a preservation and storage agreement that there will be an attempt at reanimation in the future, even though there is no guarantee that it will work. For example, if a contract stated, ‘there is no guarantee of reanimation or of reanimation being successful’ this could still imply or be understood to mean that ‘an attempt will be made if the science indicates that an attempt is possible’. It would be for the court to determine whether such a term ought to be implied to give effect to the parties’ intentions at the time of contracting.^165^ Were such a term not implied, it seems that it would be possible for cryonics organisations to effectively hold cryons ‘for ransom’, demanding additional sums for reanimation from the estate, for example, where it still exists. This also raises concern about the potential for exploitation in the way that these agreements for cryonic preservation storage are drawn up.

## VI. CONCLUSION: CRYOPRESERVATION IS A CURRENT LEGAL PROBLEM

While the future reanimation of cryons seems science fiction, people are entrusting their bodies to cryonics organizations in the hope that one day this might be possible. The number of people doing so is increasing and while cryonics is not, or ever likely to be, a common choice, it raises many important and contemporary legal problems. It is also likely that some of the conflicts and potential problems we have described in this paper will occur in this jurisdiction. As Jackson J noted in the only case concerning cryonics to come before an English court thus far,

‘I am acutely aware that this case gives rise to a large number of issues that cannot be investigated in the course of a hearing of this kind’.

Jackson J also noted the need for regulation of cryonics, and this article has explored potential disputes and the important legal questions that could arise, illustrating why regulation should be considered. We have demonstrated that in cryonics arrangements and agreements, the person who is the consumer—opting for cryonics services in the event of their death—is vulnerable to potential exploitation before and after death. Particular concerns arise about what this means in terms of the relative power of cryonics organizations, which, as we illustrated, may be making risk-free arrangements with individuals for services that they are not penalized for not performing. Equally, there is the concern that the services they perform on bodies render those bodies into property over which there might be genuine disputes about ownership. Most importantly, the potential lack of remedy for non-preservation or negligent preservation also puts the next of kin of the preserved person in a vulnerable position, potentially suffering serious distress without remedy. For some, these issues may be grounds to debate whether cryonics should be prohibited.^166^ We do not go this far in this article, since we have emphasized the importance of respecting the wishes of the dying and the dead and because these are all issues that *might* be addressed with proper regulation.

We hope that the painful practical disputes we have described do not arise, but even if some of the concerns discussed do not materialise, our analysis is valuable. Cryonics is a good legal imaginary for reflecting upon the substance of ‘private rights’ postmortem. There are some circumstances where we talk and think about the rights of a person premortem to make decisions about what happens after their death. Such reflections and conversations are profoundly important, but there are all sorts of conflicts that can arise after the fact that raise questions about how far we respect the premortem wishes of the dead, and in what circumstances the wishes of the dead may be disrespected or perhaps justifiably compromised. If the rights and wishes of the dead in this context are to be afforded greater legal protection, who inherits the rights or obligations in enforcing them, and to what extent should the law interfere if those obligations are not fulfilled? How do we navigate conflicts between the living about the dead, or between the living and the now-dead? Our examination of current potential conflicts associated with cryopreservation has illuminated the precarity of this area of law. While a person’s wishes about what happens to their money and property are afforded legal protection, their wishes about what happens to their body after death are treated as a far less important matter in English law (and many other jurisdictions). The option of cryopreservation also highlights the vulnerability of those seeking cryonic preservation, and their family, who may suffer as a result of the dream of immortality sold by cryonics organizations.

## Footnotes

1 Melissa Lawford, *How Much Would You Pay to Come Back From the Dead? The Starting Price is £60k,* (2021) https://www.telegraph.co.uk/money/consumer-affairs/much-would-pay-come-back-dead-starting-price-60k/ (accessed Dec. 6, 2021).

2
*Re JS (Disposal of Body)* [2016] EWHC 2859 (Fam).

3
*Id.* at 30–32 per Jackson J.

4
*Id.* at 69.

5 For example, Ole Moen, *The Case for Cryonics* 41 J Med Ethics 677 (2015); F. Minerva, the Ethics of Cryonics: is it Immoral to be Immortal? (Palgrave Macmillan 2018).

6 Heather Conway, *Frozen Corpses and Feuding Parents: Re JS Disposal of Body*, 18 MLR 132, 135 (2018).

7 For further discussion about the value of speculating about future technologies see: Claire Horn and Elizabeth Chloe Romanis, *Establishing Boundaries for Speculation About Artificial Wombs, Ectogenesis, Gender, and the Gestating Body*, in A Jurisprudence of the Body (C. Dietz et al. eds., 2020).

8 Nils Hoppe, *Justice cryogenically delayed is justice denied?* (2016) https://blogs.bmj.com/medical-ethics/2016/11/18/justice-cryogenically-delayed-is-justice-denied/ (accessed Dec. 7, 2021).

9 This is name given to the process of preventing tissue damage caused by freezing by replacing blood in the human body with human ‘antifreeze’ solutions designed to preserve organs.

10 It is thought that this technology will enable the successful reanimation of ‘cryonic suspension patients’ in the future. This is because this technology will be able to ‘cure’ the ailments that were causing a person’s death before they were preserved. A lot less is known about nanotechnology that is necessary to be certain that it holds the capacities anticipated. Also little is known about how preserved bodies can be ‘reanimated’ safely.

11 Otherwise, there is the potential for the person undertaking cryopreservation to be considered homicide, or even grievous bodily harm.

12 Alcor Life Extension Foundation, ‘*Frequently Asked Questions*,’ (nd) https://www.alcor.org/what-is-cryonics/#toggle-id-2 (accessed Aug., 2023).

13 Alcor Life Extension Foundation, *What is cryonics,* (nd) https://www.alcor.org/what-is-cryonics/ (accessed Aug., 2023).

14 Sanka Bhattacharya, *Cryoprotectants and Their Usage in Cryopreservation Process,* in Cryopreservation Biotechnology in Biomedical and Biological Sciences, (Yusuf Bozkurt ed., 2018), at 8.

15 Alcor, *supra* note 12.

16 We use this term to refer to the (deceased person that is the) subject of cryonic preservation.

17 Alcor Life Extension Foundation*, Alcor Membership Statistics*, (nd) https://alcor.org/AboutAlcor/membershipstats.html (accessed Dec. 8, 2021; Cryonics Institute, *Welcome to Cryonics Institute,* (nd) https://www.cryonics.org/ci-landing/ (accessed Dec. 8, 2021).

18 Trans Time, (nd) http://www.transtime.com/index.html (accessed Dec. 8, 2021).

19 For example, Cryocare Foundation (nd) http://www.cryocare.org/ (accessed Dec. 7, 2021).

20 Kriorus (nd) http://kriorus.ru/en (accessed Dec. 7, 2021).

21 Alcor Life Extension Foundation, *How Much Does Cryopreservation Cost?* (nd) https://www.alcor.org/membership/#toggle-id-1 (accessed Jan. 5, 2023).

22 Alcor provides neurocryopreservation as an option because they hold that preserving the brain is both necessary and sufficient for the revival of the person in the future.

23 For details of their organization see; cryonics-uk.org

24 See David Brown, *Body Freezing Group Wins Appeal to Preserve Its Status as a Charity*, (2018) https://www.thetimes.co.uk/article/body-freezing-group-wins-appeal-to-preserve-its-status-as-a-charity-tzc57bdfg (accessed Dec. 15, 2022).

25 Our addition, although this was flagged by Jackson J earlier in the same passage.

26
*Re JS*, *supra* note 2, at 12.

27
*Id.* at 68(2).

28 Alcor, on their website, explains their belief that death is a process that can be ‘paused’: See Alcor, *supra* note 13.

29 Alcor describes itself as a ‘Life Extension Foundation’.

30 See Cryonics Institute. *supra* note 17.

31
*Re A* [1992] 3 Med LR 303.

32
*Airedale NHS Trust v Bland* [1993] 1 All ER 821.

33 Academy of Medical Royal Colleges, *A Code of Practice for the Diagnosis and Confirmation of Death,* (2008) https://www.aomrc.org.uk/wp-content/uploads/2016/04/Code_Practice_Confirmation_Diagnosis_Death_1008-4.pdf (accessed Dec. 15, 2022), 11.

34
*Re M (Declaration of Death of Child),* per MacFarlane P at 91.

35
*Barts Health NHS Trust v Dance and others* [2022] EWFC 80, per Hayden J [2].

36 Academy of Medical Royal Colleges, *supra* note 33, at 35.

37 Moen, *supra* note 5.

38 For example, Spain. For discussion about inheritance rights and interests see Iain Brassington, *On Rights of Inheritance and Bequest* 23 J Ethics, 119 (2019).

39
*Re JS, supra* note 2; *Burrows v HM Coroner for Preston* [2008] EWHC 1387 (QB).

40 Conway, *supra* note 6.

41
N. Cantor, After We Die; the Life and Times of the Human Cadaver, 59 (Georgetown Press 2010).

42
*Burrows v HM Coroner for Preston* [2008] EWHC 1387 (Admin).

43
*R v Stewart* (1840) 12 Ad and EI 773, 113 ER 1003 (QB). This right, however, could be denied for certain people such as convicted murderers or those who died by suicide. For a useful discussion see Thomas Muinzer, *The Law of the Dead: A Critical Review of Burial Law, with a View to its Development,* 34 OJLS 791 (2014).

44
*R v Newcomb* (1898) 2 CCC 255.

45 Administration of Estates Act 1925, s.46 and Non-Contentious Probate Rules 1987, rule 22(1).

46 The Public Health (Control of Disease) Act 1984.

47 For a detailed discussion of the law see H. Conway, the Law and the Dead, Ch 3 (Routledge 2016).

48 The Law Commission, *A Modern Framework for Disposing of the Dead*, (nd.) https://www.lawcom.gov.uk/project/a-modern-framework-for-disposing-of-the-dead/ (accessed Dec. 15, 2022).

49 Civil Code, Chapter II Respect for the Human Body, Article 16-1-1

50
*Re JS*, *supra* note 2: Jackson J confirmed that, ‘…what JS wants does not seem to be illegal’, and that no offense would be committed, at 16.

51 For example, Lasting Power of Attorney per the Mental Capacity Act 2005.

52 Michael Darwin et al., ‘*Is That What Love Is? The Hostile Wife Phenomenon in Cryonics*,’ (2009) https://web.archive.org/web/20090308065150/http://www.depressedmetabolism.com/is-that-what-love-is-the-hostile-wife-phenomenon-in-cryonics/ (accessed Dec. 15, 2022). We note that much of the specific discussion on this discussion post is both essentialist and misogynist.

53 Richard Sandomir, *Baseball: Williams Children Agree to Keep Their Father Frozen,* (2002) https://www.nytimes.com/2002/12/21/sports/baseball-williams-children-agree-to-keep-their-father-frozen.html (accessed Dec. 15, 2022).

54
*Williams v Williams* (1882) 20 Ch D 659.

55 Conway, *supra* note 47, at 67; NB where the person with the right to possession for burial does not exercise their duty to do so, the Court can exercise its inherent jurisdiction to permit the local authority, for example, to do so per *Re K (A Child) (Disposal of Body: Court’s Power to Authorise)* [2017] 4 W.L.R. 112.

56
*Buchanan v Milton* [1999] 2 FLR 844, at 846–847. It is important to acknowledge, though this was not properly done in the decision, that this case (though often discussed as a case of dispute between adoptive family and birth family) concerned a person who as a child was improperly removed from his birth family as a part of the ‘stolen generation’ of indigenous children in Australia.

57
*Anstey v Mundle* [2016] EWHC 1073 (Ch). We note the only exception is in relation to very sensitive cases where there is no next of kin, e.g. Ian Brady’s request for music to be played at his funeral was denied and the court ordered that his remains must be disposed of secretly (rather than being placed in the custody of Brady’s solicitor, Robin Makin) in order to avoid the site of disposal becoming known to the public (*Oldham MBC and Tameside MBC v Robin Makin, X, and Sefton MBC* [2017] EWHC Case no; HC-2017-002064 ch).

58 Cantor, *supra* note 41, at 55.

59 (1930) 65 OLR 586.

60 (1993) 104 DLR (4th) 384.

61 [2008] EWHC 1387 (Admin).

62 Ministry of Justice, *Guide to Coroner Services for Bereaved People,* (2020) https://assets.publishing.service.gov.uk/government/uploads/system/uploads/attachment_data/file/363879/guide-to-coroner-service.pdf (accessed Dec. 15, 2022).

63 Coroners and Justice Act 2009.

64 Note that only hospital autopsies require the consent of the next of kin. Where a person dies in a clinical setting and there are no suspicious or unexplained circumstances but doctors wish to conduct an autopsy, the family must consent to such an autopsy. This is set out in the Human Tissue Act 2004.

65 Coroners and Justice Act, s.40(1).

66 See Conway, *supra* note 47, at 16–18.

67
*Id.* See also, *R (on the application of Rotszein) v HM Senior Coroner for Inner North London* [2015] EWHC 2764 (Admin), which concerned an article 9 ECHR challenge.

68 We are grateful to Dr Irina Sakharova for discussions on this point.

69 John Adams and Roger Brownsword, *The ideologies of contract*, 7 LS 205, 208 (1987).

70 Roger Brownsword, *The Limits of Freedom of Contract and the Limits of Contract Theory*, (1995) 22 Journal of Law and Society 259–273, 263.

71
*Id*. at 264.

72 We return to remedies later in this section.

73
*Robinson v Davison* (1871) LR 6 Ex 269

74
*Beswick v Beswick* [1967] UKHL 2

75 Tamar Lapin, *Cryogenics Company Only Froze Man’s Head Instead of His Whole Body: Suit*, (2018) https://nypost.com/2018/09/10/cryogenics-company-only-froze-mans-head-instead-of-his-whole-body-suit/ (accessed Dec. 6, 2021).

76 Tyler Hayden, *In Cryonics Lawsuit, Son Fights for Father’s Frozen Head*, (2019) https://www.independent.com/2019/04/17/in-cryonics-lawsuit-son-fights-for-fathers-frozen-head/ (accessed Dec. 6, 2021).

77 This point was raised by an anonymous reviewer.

78 Consumer Rights Act 2015, s.2 (2)–(3).

79
*Id.,* s.49(1).

80 Consumer Rights Act 2015, s.54(3)(b).

81
*Johnson v Agnew* [1980] AC 367.

82 As we noted above, this might be an important factor to consider in a normative analysis of whether the law ought to recognize cryonics contracts.

83 Consumer Rights Act 2015, s.54(3)(a).

84
*Id.,* s.54(3)(b).

85
*Id.,* s.54(7)(a).

86
*Id.,* s.54(7)(c)—though note specific performance is unlikely to be a possible remedy in cases where a cryonics organization has breached a contractual arrangement by failing to preserve a body.

87
*Id.,* s.54(7)(f).

88
*Id.,* s.55(3)—where repeat performance is impossible, specific performance would also be precluded for the same reasons.

89
*Id.*, s.56(3)(a).

90 For example, the body is not in a suitable condition because of the manner of death.

91
*Robinson v Harman* (1848) 154 ER 363.

92
*Id.*

93
*Jarvis v Swan Tours Ltd* [1972] EWCA 8.

94 D Harris et al., *Contract Remedies and the Consumer Surplus* 95 LQR 581 (1979).

95
*Watts v Morrow* [1991] 1 WLR 1421, per Bingham LJ.

96
*Id.*

97
*Farley v Skinner* [2001] UKHL 49, per Lord Scott.

98
*R v Price* (1884) 12 QBD 247.

99 Ernest Partridge, *Posthumous Interests and Posthumous Respect*, 91 Ethics 243 (1981).

100
*Johnson v Agnew, supra* note 78.

101 For example, *Chaplin v Hicks* [1911] 2 KB 786.

102 For example, *Allied Maples Group Ltd v Simmons & Simmons* [1995] 1 W.L.R. 1602.

103
*Chaplin supra* note 97.

104
*Id.,* per Moulton LJ at 789.

105
*Id.,* per Williams LJ at 792

106
*Id.,* per Moulton LJ at 795.

107
*Id.,* per Williams LJ at 792–93.

108
*Id.,* per Farwell LJ at 799.

109
*Ruxley Electronics and Construction Ltd v Forsyth* [1995] UKHL 8.

110
*Addis v Gramophone Co Ltd* [1909] AC 488.

111
*Robinson v Harman*, *supra* note 85.

112
*Farley, supra* note 93.

113 Hayden, *supra* note 67.

114
*Beswick, supra* note 72.

115
*Jackson v Horizon Holidays* (1975) 1 WLR 1468.

116
J O’Sullivan, O’Sullivan & Hillard’S the Law of Contract, 140 (Oxford University Press 2020), 140.

117
*Jackson, supra* note 111, at 1473.

118
*Id.*

119 Contracts (Rights of Third Parties) Act 1999, s.1(1)(a).

120
*Id.,* s.1(1)(3).

121
*Id.,* s.1(6).

122 We discuss this possibility in detail in the next section.

123
*Alcock v Chief Constable of West Yorkshire* [1991] 4 All ER 907, HL. There must be a close tie of love and affection. Parents, children and spouses will be presumed to be sufficiently close but for other relationships the claimant must prove that they enjoyed a close, affectionate bond with the deceased/primary victim.

124
*White v Chief Constable of South Yorkshire* [1998] 3 WLR 1509, per Lord Steyn.

125
*McLoughlin v O’Brian* [1983] 1 AC (HL).

126
*Alcock*, *supra* note 119.

127 See *Bourhill v Young* [1943] AC 92.

128
*Id.*

129 [2003] EWCA Civ 697.

130
*Gammon v Osteopathic Hospital of Maine* 534 A.2d 1282 (Me. 1987).

131 Kevin Bry, *Genuinely Distressing: Illinois’ Failure to allow a Cause of Action for Emotional Injuries Caused by Negligent Mishandling of a Corpse*, 23 J Marshall L Rev 353 (1990).

132
*R v Kelly and Lindsay* [1998] 3 All ER 741 (CA). See M Quigley, Self-Ownership, Property Rights, and the Human Body (Cambridge University Press 2018).

133
*Dobson v North Tyneside Health Authority* [1996] 4 All ER 474.

134
*Doodeward v Spence* (1908) 6 CLR 406.

135
*Id.,* 414 per Griffiths CJ.

136
*Kelly, supra* note 130.

137 Andrew Grubb, *I, Me, Mine: Bodes, Parts and Property*, 3 Med Law Int 299, 310 (1998).

138
*Dobson, supra* note 131.

139
J. Wall, Being and Owning: the Body, Bodily Material and the Law, 16 (Oxford University Press 2015).

140
*Kelly*, *supra* note 130, at 631.

141 [2004] EWHC 644 (QB).

142
*Id.* at 148.

143
*supra* note 132, at 414.

144 See context section for detail.

145 We are grateful to Dr Irina Sakharova for this point.

146 [2009] EWCA Civ 37.

147 For discussion see Nils Hoppe, *Yearworth et al. v North Bristol NHS Trust [2009] EWCA Civ 37,* (2010) https://www.hoppe.legal/yearworth-et-al-v-north-bristol-nhs-trust-2009-ewca-civ-37/ (accessed Dec. 15, 2022).

148 Yearworth (n154), [28] per Judge CJ.

149
*Id*. at 28.

150
*Id.* at 45.

151
*Id.* at 45.

152 Human Fertilisation and Embryology Acts 1990 and 2008.

153
*Supra* note 159.

154
*Supra* note 141.

155
*Re St Andrew’s Churchyard, Alwalton* [2012] P.T.S.R. 479.

156
*Id.*, per Gregory Jones Dep Ch at 51.

157 ‘To accede to such a request would not only create a precedent in terms of undermining the status of the permanence of burial’ *Id.* at 44.

158
*Supra* note 140.

159 Grubb, *supra* note 145, at 308.

160
*Supra* note 154.

161 This was the case in all of the cases where the work and skill exception was applied that we are aware of.

162 We are grateful to Dr Irina Sakharova for raising this point with ECR.

163 Sheelagh McGuiness and Margot Brazier, *Respecting the Living means Respecting the Dead Too*, 28 OJLS 297 (2008).

164 For example, KrioRus: Yana Pashaeva, *A Divorced Couple Is Fighting Over Frozen Dead Bodies,* (2021) https://slate.com/technology/2021/09/cryonics-dead-bodies-russia-frozen-kriorus.html (accessed Jan. 5, 2022).

165
*Shirlaw v Southern Foundries Ltd* [1939] 2 KB 206.

166 We are grateful to an anonymous reviewer for encouraging us to think about this.

